# Gluten-Free Product Contribution to Energy and Macronutrient Intakes in Spanish Children and Adolescents with Celiac Disease

**DOI:** 10.3390/foods11233790

**Published:** 2022-11-24

**Authors:** María Purificación González, Catalina Ballestero-Fernández, Violeta Fajardo, María Achón, Ángela García-González, Elena Alonso-Aperte, Natalia Úbeda

**Affiliations:** Food and Nutrition in Health Promotion Research Group (CEU-NutriFOOD), Departamento de Ciencias Farmacéuticas y de la Salud, Facultad de Farmacia, Universidad San Pablo-CEU, CEU Universities, Urbanización Montepríncipe, 28668 Boadilla del Monte, Spain

**Keywords:** gluten, gluten-free, celiac disease, children, adolescents

## Abstract

Gluten-free products (GFP) are a good choice for the replacement of cereals when following a gluten-free diet due to celiac disease (CD). However, commercial GFP are made with highly refined flours and may contain more fat, sugar, and salt, and less fiber and micronutrients than gluten-containing analogues, thus challenging the nutritional adequacy of the diet. The aim of this study is to assess the contribution of GFP to the diets of children and adolescents with CD. Food intakes were assessed in a cross-sectional study on 70 children and adolescents with CD (aged four to 18, 50% females), using three 24-h dietary records. GFP consumption reached 165 g a day and comprised mostly bread and fine bakery ware, followed by pasta. GFP contributed with a high percentage (>25%) to total energy, carbohydrates, fiber, and salt daily intakes and, to a lesser extent (<20%), to fat (including saturated fat), sugars and protein. Contribution of homemade products was testimonial. GFP contribution to total energy intake is significant and, consequently, relevant to the nutritional adequacy of the diet. Children and adolescents with CD could benefit from fat, saturated fat, and salt reduction, and fiber enrichment of processed GFP.

## 1. Introduction

Celiac disease (CD) is a systemic immune disorder triggered by an aberrant response to gluten proteins that causes progressive atrophy of the intestinal villi of genetically susceptible individuals, resulting in impaired intestinal absorption and malnutrition, although in many cases the disease is asymptomatic [1]. The prevalence of CD in European and American populations of European descent is 1% [2].

The only effective therapy for CD is to follow a rigorous and lifelong gluten-free diet, which regenerates intestinal villi and restores adequate nutrient absorption, although only 1/3 of adults have normal villous architecture (a healthy, healed intestine) after two years, and 2/3 after five years on a gluten-free diet [3]. However, diagnosis during childhood and less severe histologic damage at diagnosis have been associated with mucosal recovery [4]. The gluten-free diet comprises food products that originally do not contain gluten, i.e., legumes, eggs, fruits and vegetables, meat and fish derivatives, and the avoidance of wheat, barley, rye, oats, and all products derived from these cereals (starch, flour, breads, pasta, etc.), as well as any product in which gluten containing ingredients are used by the food industry [5].

Some studies indicate nutritional imbalances in different celiac populations following a gluten-free diet in terms of macro and micronutrient intakes because of a bad choice of foods and gluten-free products (GFP) [6]. Nutritional studies undertaken in children and youngsters, some of which have compared with a control group without the disease, have shown that CD patients consume more lipids (especially saturated), protein and simple carbohydrates, but less fiber and micronutrients such as iron, calcium, zinc, magnesium and vitamin D, folates and other B-vitamins than recommended [7,8,9,10,11,12,13,14,15,16,17,18,19,20,21,22,23,24], even if adherence to the gluten-free diet is long-term (more than one year). Childhood and adolescence carry unique issues pertaining to the diagnosis and management of celiac disease, because of the high nutritional requirements for growth during these periods. Moreover, dietary habits acquired in childhood and adolescence prevail in adulthood.

Different authors have proposed that nutritional deficiencies in CD patients may be due to GFP, which are made with highly refined flours and extra amounts of fat and sugar to obtain a texture resembling the typical and unique viscoelastic properties of wheat [25,26]. Gluten-free manufactured products are generally high in fat to improve their presentation and palatability [13], and recent articles conclude that GFP often have a greater carbohydrate and lipid content, but less protein than their gluten containing equivalents [27,28,29,30]. Macronutrient analysis of GFP commercially available in Spain has evidenced that 25.4% of the products could be labelled as a source of fiber, and many presented very high contents of energy (33.5%), fats (28.5%), saturated fatty acids (30.0%), sugars (21.6%), and salt (28.3%) [31]. Folates and other B-vitamins were also present in significantly lower amounts than in their gluten-containing counterparts [32,33,34,35]. According to Allen and Orfila [36], only 5% of gluten-free breads were fortified with all four mandatory fortification nutrients (calcium, iron, nicotinic acid or nicotinamide, and thiamine) in the United Kingdom, and only 28% of gluten-free breads were fortified with calcium and iron. Fortified GFP represent only 10% of gluten-free staple foods in Europe, because different starches are used as main ingredient and that makes it difficult to implement a common fortification strategy [37].

Furthermore, the use of raw materials such as unenriched rice or maize refined flours, gums, or enzymes in GFP formulation could lead to a different composition as compared to their gluten-containing homologues [38]. GFP commercially available in Spain are primarily composed of rice and/or corn flour, and only a 28% of products are supplemented with a low quantity of quinoa, soy and other legumes, or other flours (oatmeal, sorghum, amaranth, teff, guar, chia, chestnut, flax, potato) [31]. Since the micronutrient content of gluten-free pseudocereals and legumes is higher than that of the gluten-free cereals [16], some authors have proposed the promotion of their use in GFP formulation [25,26,35]. Breakfast cereals were the group with the most frequent inclusion of alternative cereals in GFP in the Spanish market [31]. Therefore, manufacturers seem to be timidly introducing the use of nutritious pseudocereal and legume flours in the formulation of GFP. In this sense, Melini and Melini [25] have also shown that an interesting trend towards some improvements in GFP composition has emerged. In this sense, although the composition in terms of fiber and sugars is highly variable among different product varieties, reports of the two last years showed more adequate levels of fiber and sugar than in the past. In fact, Larretxi et al. [39] have reported an equivalent fiber and resistant starch content, which was even higher in breads, compared with their homologues. The increased fiber content and improved technological processes have positively affected the glycemic responses from these goods [32].

The insufficient labelling description and narrow availability of nutritionally balanced products and brands for the population affected with CD may limit to a greater or lesser extent the ability to follow a balanced diet. The question is whether the consumption of these products by the celiac population is sufficient to cause the nutritional imbalances, or whether it is due more to poor food choices from other food groups. Bearing this in mind, our aim in this study is to assess the contribution of GFP to the diet (energy and nutrient content) of a group of children and adolescents, both genders, with CD in Spain.

Only by analyzing the consumption of GFP and the amount of critical nutrients they provide in relation to the total diet will it be possible in the future to carry out correct and effective nutritional education for this population group, offering an appropriate choice of foods, both fresh and processed. In this sense, it is important that children and young people with CD become aware of the importance of reading the labelling in terms of ingredients and nutritional composition. The data in this study may also be useful for the food industry in reformulation or fortification strategies on GFP.

## 2. Participants and Methods

### 2.1. Participants

Sample size was calculated for an initial cross-over study comparing dietary habits and nutritional status of children and adolescents with and without celiac disease (CD) [18]. Considering previous studies carried out in Spain and a confidence interval of 95%, an alpha error of 5%, a power of 80%, a case-control ratio of 1:1 and a predicted loss rate of 20%, sample size was estimated in 110 subjects by group. Finally, 75 individuals were recruited which conducted to a final error fixed in 6%.

Participants were children and adolescents diagnosed with CD between 4 and 18 years old, affiliated to the Celiac and Gluten Sensitive Association in Madrid, Spain (Asociación de Celiacos y Sensibles al Gluten de Madrid). To be included in the study, participants needed a previous confirmed medical diagnosis of CD, and should be following a gluten-free diet for over more than a year, without taking nutritional supplements, and not suffering associated diseases. Immunoglobulin A (IgA) anti-tissue transglutaminase antibodies (IgA-tTG) were analyzed in blood samples from all participants to screen for adherence to the gluten-free diet. Before enrolling as participants, volunteers and guardians or caregivers were informed and provided their written consent Anonymity was guaranteed. The project was conducted in accordance with legal requirements and guidelines for good clinical practice, as well as the World Medical Association Declaration of Helsinki on Ethical Principles for Medical Research involving Human Subjects (revised in October 2008). The protocol was approved by the Ethics Committee for Human Studies in Universidad San Pablo-CEU (Authorization number 102–15).

### 2.2. Food Habits and Nutrient Intakes

As detailed in previous published studies [18] in the first visit, a trained dietitian interviewed the participants for recalling: personal data, family history of disease, and medication use. Following the recommendations by the European Food Safety Authority [40], the diet was assessed using three 24-h dietary records. The first one was carried out during the visit, by the dietitian and relatives helped the volunteers to complete the records when necessary. Two more 24-h dietary records were assessed via phone call with a time difference interval of one month. One of the three 24-h dietary records was taken on a Sunday or a holiday. Volunteers who consumed commercial gluten-free products (GFP) were asked to record the specific brand. Data on composition, as provided by the manufacture’s label, were recorded, to build a composition database on commercial GFP and use the data for the assessment of nutrient intake. The GFP composition database is available at the Universidad San Pablo-CEU institutional repository [41]. It must be noted, however, that data provided by manufacturers were limited to those under European regulation, i.e., energy value, amounts of fat, saturated fat, carbohydrates, sugars, protein, and salt. Labels do not record data on micronutrient composition, therefore, data on micronutrient intake from these products were not quantified. The records were analyzed using the DIAL^®^ software, version 3.15 (Alce Ingeniería, Madrid, Spain) to transform food intake into energy and nutrient consumption. 

### 2.3. Statistical Analysis

Statistical analysis was performed using IBM SPSS^®^ Statistics, version 27.0 (Somers, NY, USA). The variables were checked for normality by the Kolmogorov–Smirnov test, and, considering the results, the data were expressed as median (percentile 25−percentile 75) or mean ± standard deviation. The comparison between the two groups (by sex or age group), was analyzed using the Mann–Whitney and Student’s *t*-tests. Level of significance was set at *p* < 0.05 for all statistical analyses. 

## 3. Results

A total of 70 Spanish children and adolescents with celiac disease (CD) participated in the study (35 boys and 35 girls, 56 aged 4–12 years old, and 14 aged 13–18 years old). They all declared themselves to have been on a gluten-free diet for more than one year, and 96.8% stated good adherence. In the immune tests, all values fell under threshold values (<6.9 U/mL) for untreated CD or accidental exposure to gluten. A great part of the sample (92.9%) consumed processed gluten-free products (GFP) three to four times a day, and only one volunteer declared not taking gluten-free substitutes. Most participants consumed processed GFP (98.5%), but only 30% of them consumed homemade GFP once a day or less. Volunteers declared taking between four to five meals a day, usually at home except for school days (zero to one meal a week outside the home), and seldom taking fast food (twice a month as the median). We have previously published data on dietary intake, food consumption patterns, and biochemical and anthropometric data from this same population group [18].

Table 1 shows daily intake of gluten-free cereal-based substitutes, both processed (commercially available) and homemade. Children and adolescents with CD in Spain consume over 165 g a day of GFP, which are mostly bread and fine bakery ware, followed by pasta. Fine bakery ware includes biscuits and pastry in similar proportions, except for adolescents, who take a smaller amount of biscuits. Intake of gluten-free breakfast cereals and cereal bars is much lower, and consumption of gluten-free pizza, prepared food products and snacks varies significantly between volunteers. Only 34 out of the 70 volunteers declared that they had been taking gluten-free savory commercial products, i.e., pizza, nuggets, nachos with cheese, tortitas, gnocchi, pie, or croquette. There are no big differences between groups based on sex and age, except for a higher consumption of biscuits among children aged four to 12, and a higher consumption of savory cereal dishes in boys and adolescents over 12. Consumption of homemade GFP is testimonial as compared to commercial products.

Table 2 shows the percentage contribution of GFP to total energy and nutrient intakes, in Spanish children and adolescents, using the nutrient information presented on the labels. GFPs contributed with a high percentage (>25%) to energy, carbohydrates, fiber, and salt daily intakes and, to a lesser extent (<20%), to fat (including saturated fat), sugars and protein. When considering age and sex (Table 3), GFP contribution to total intakes is higher in boys than girls for all nutrients analyzed, although only statistically significant in the case of saturated fat. There seems to be no effect of age; and GFP percentage contribution to energy, macronutrient, fiber, and salt intakes is similar between children aged four to 12 and adolescents over 13.

Table 4, Table 5, Table 6, Table 7, Table 8 and Table 9 provide an in-depth analysis of the contribution of each type of GFP to energy and nutrient intakes. The GFP with significantly higher contributions to total energy intake were bread and fine bakery ware, which accounted in similar proportions for 70% of the total energy provided by GFP (Table 4). In the case of fat and saturated fat (Table 5), fine bakery ware was the greatest contributor, providing a significantly higher amount—three-fold—as compared to the second greatest contributor, bread. All GFP provide carbohydrates, but fine bakery ware contributes with 59% of all sugars provided by GFP (Table 6). Percentage contribution of GFP to total protein intake (Table 7) is low in all cases. Bread is the most important contributor to fiber intake within the GFP, accounting for 56% of all fiber provided by GFP (Table 8). On the other hand, salt is provided mainly by bread, which accounts for 52% of the salt provided by GFP (Table 9). Regarding gender, savory cereal dishes contribution to total energy, saturated fat, carbohydrates, sugars, and salt was higher in boys as compared with girls. Fine bakery ware provides a lesser amount of fiber in adolescents as compared with younger children.

**Figure 1:**
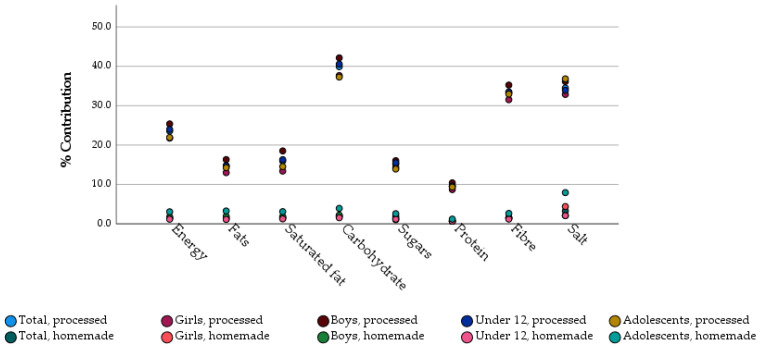
Contribution of processed and homemade gluten-free products to the diet (energy and nutrient content) of Spanish children and adolescents with celiac disease depending on gender and age. Data are expressed as average ± standard deviation. There are statistics differences (*p* < 0.01) in the contribution of energy and nutrients to total diet between processed vs. homemade in all cases.

Finally, we assessed gluten-free homemade products and gluten-free commercial products separately. In this context, we observed a minimal contribution of homemade products, being most energy and nutrients coming from processed products (Figure 1). Homemade GFP accounted for 1.5% of total energy intake, whilst processed commercial products provided 23.4% of total energy. No differences in consumption of homemade or processed commercial products were detected depending on age or gender.

## 4. Discussion

To our knowledge, this is the first study to assess, in a comprehensive way, the contribution of gluten-free products (GFP) to energy and macronutrient intakes in Spanish children and adolescents with celiac disease (CD), and the only one that, in addition, considers both homemade and commercial products. GFP contributed with a high percentage to energy, carbohydrate, fiber, and salt daily intakes and, to a lesser extent to fat (including saturated fat), sugars and protein. Boys tended to consume a higher amount of GFP as compared with girls, but GFP contribution to total diet was only significantly higher for boys in the case of saturated fat. However, age seemed to make no difference, with GFP contribution to daily energy and macronutrient intakes being similar in children under 12 years old and adolescents over 12. In all cases, we observed a minimal contribution of homemade products, and most energy and nutrients came from processed commercial products. 

Our results are slightly different to those shown by Zucotti et al. [13] in their cross-sectional study on children with CD as compared to controls, in which commercially available GFP accounted for 36.3% of daily total energy intake and 18% of total protein-derived energy in Italian children. In this same research [13], almost half (49.5%) of the total carbohydrate intake and the majority (77.0%) of the 53.2% carbohydrate-derived daily energy was derived from GFP, results which are higher than those found in our study (almost 40% of total carbohydrates and 40% of carbohydrate-derived energy are provided by GFP). In the case of fat, our results are similar to those shown in Italian children, where GFP contributed a median of 12.9 g per day of fat intake. On the other hand, Larretxi et al. [39] have revealed that the main contributors to the fiber intake of Spanish adult and pediatric participants following a gluten-free diet are GFP (~50%), especially breads, but fiber intake was still slightly below recommendations. In our study, GFP contribution to daily fiber intakes is lower (~36%).

According to a cross-sectional study using a representative sample of Spanish population [42], grains (i.e., grains and flours, bread, breakfast cereals, cereal bars, pasta, and bakery and pastry) are the main contributors to energy, carbohydrate, and fiber intakes, providing up to 30%, 49% and 47% of total daily intake in children (aged 9–12 years), and up to 31%, 51% and 49% of total daily intake in adolescents (aged 13–17 years). This same study [42] showed that the main contributors to protein intake are meat and meat products, followed by grains and grain-based products, and milk and dairy products, contributing, all together, to about 72% of the protein intake in children and adolescents. Finally, grains were also the fourth contributor to lipid intake (12%) in children and adolescents, providing a significant amount of saturated fat from bakery wares and pastry in children (11%) and 15% of total sugar intake [42]. In the present study, GFP provided 25% of total energy, an amount which is close to that described for all grains in general population in Spain with the same age. Moreover, GFP contribution to energy daily intake is in the range of a healthy breakfast, which according to dietary recommendations in Spain should contribute around 20–25% to the total daily energy intake [43]. Therefore, GFP contribution to total energy intake in children and adolescents with CD may be considered significant and, consequently, relevant to the nutritional adequacy of the diet. 

Our data, taken together, show a high consumption of products marketed and labelled as gluten-free, the vast majority of which are processed. Although some authors have described an incipient reformulation of these products [31], especially concerning fiber and types of fat, and no differences in fiber content between GFP and their gluten-containing counterparts has been described [39], high GFP consumption could raise several problems, both nutritionally and otherwise, since the low nutritional quality of these products has been highlighted in various studies [25,26]. 

From the nutritional point of view, grains and cereal-based products are staple foods in Mediterranean type diets. Because of a different composition of GFP as compared to regular counterparts, gluten-free diets may be low in fiber and rich in fat, saturated fat, and salt [25,44]. In the case of fiber, GFP in our study provided less fiber (30%) than regular grains in Spanish children and adolescents [42]. Gluten-free bread was the main contributor. Nonetheless, GFP plus other naturally gluten-free grains i.e., rice and corn, and other food sources contributed to a total dietary fiber intake which, as a median value, was adequate in children (18 g/day), but low in adolescents (17 g/day), according to dietary reference values from the European Food Safety Authority (EFSA) i.e., 14–16 g/day for children between four and 10 years old, and 19–21 g/day for adolescents between 11 and 17 years old [45]. This may be due also to a higher intake of fruits in boys with CD as compared to controls, as we have described before in this same study group [18]. 

As compared to regular grains, GFP contribution to total and saturated fat intakes in our study was higher than that of all grains in healthy children and adolescents consuming a regular diet in Spain (16% vs. 13% for total fat and 17% vs. 13% for saturated fat), as published in the ANIBES study [42]. The GFPs that most contributed fat and saturated fat were fine bakery ware. Moreover, GFP contribution was particularly high in males and for saturated fat (17%), posing a worrying situation for children and adolescents following a gluten-free diet. Other authors have already stated that following a gluten-free diet increases the risk of cardiometabolic related pathologies such as obesity [46,47] or cardiovascular disease [48]. On this basis, it seems reasonable to propose a fat and saturated fat reduction in GFP formulation, especially in the case of fine bakery ware.

GFP were also important contributors to total sugar intake, especially in the diets of children on a gluten-free diet under 12 years old and boys, where they contributed to almost 18% of total sugar intake. This is a 30% higher amount as compared to Spanish children, whose diets include grains that provide 13% of total sugar intake, as described in the study by Ruiz et al. [42]. Children under 12 years old on a gluten-free diet in our study were consuming 17% of total energy from sugars [18], although only 2.6% of total calories were coming from sugars in GFP. A young population group with CD in Spain (aged 10 to 23) also reported higher consumption of added sugar and total fat than healthy individuals [14]. According to recommendations [49], the intake of added and free sugars should be as low as possible in the context of a nutritionally adequate diet. Thus, children, especially boys, could also benefit from sugar content reduction in their diets. Nonetheless, GFP do not seem to contribute decisively to total sugar intakes in our study. 

GFP provide only 10% of protein daily intake, which is only half the contribution of all grains to protein intakes in healthy children and adolescents [42]. GFP are known to contain a lower amount of protein as compared to gluten-containing counterparts [27,28,29,30], due to gluten extraction or to the use of refined flours and starches in their formulation. Nonetheless, protein intake in Spanish children and adolescents with celiac disease is not below that of healthy controls [18], and protein intakes in Spain are excessive and nutritionally unnecessary in healthy individuals [42].

GFP contribution to daily salt intake reached 38% as a median value, with slightly higher contributions in boys and adolescents over 12 years old. High salt content has been described as one of the key inadequacies of GFP, besides high fat and low protein contents [25]. In Spanish population, main dietary sources of sodium are meat and meat products and cereal and grains, accounting for 53% of sodium consumption [50]. According to our data, gluten-free cereal products are also primary contributors to sodium/salt intakes, in the same order of magnitude as all grains, and bread plays a leading role. Moreover, sodium intakes in Spain in general population are above recommendations, and significantly higher amongst children and adolescents when compared to adults and the elderly [50]. Therefore, salt reduction in GFP could be an effective intervention for health promotion and disease prevention. In fact, several agreements between the Spanish Health Administration and the food industry have prompted manufacturers to decrease salt addition to foods [51] and to bread [52], as a main contributor to sodium intakes, not only because of its salt content, but also and mainly because of the frequency of consumption. Unfortunately, special products such as commercial GFP designed for consumers with CD fall out of the scope of these initiatives. 

Although the contribution of GFP to salt intakes is high, data on salt and sodium intakes should be taken cautiously since there are specific difficulties in the estimation of this nutrient. In our study, we quantified sodium coming from regular foods using data from Spanish Food Composition Tables on the DIAL^®^ software version 3.15, and salt in GFP as presented by manufacturers in the label. Therefore, we have not quantified discretionary salt use (salt added during cooking or at the table). All data on sodium have been converted to salt (1 mg sodium = 2.4 mg salt).

In our view, there are several alternative ways to improve the nutritional quality of the gluten-free diet, apart from GFP reformulation to reduce the content of critical nutrients. For example, some authors have proposed fortification of GFP. Cyrkot et al. [33] exposed the fact that gluten-free folate-rich foods represented <15% of all household food purchases, and 69% of children had low folate intakes. Along the same line of conclusions, Larretxi et al. [35] proposed GFP fortification with folate and biotin to prevent the deficiencies observed in the gluten-free diet, at least in the case of pediatric CD. These findings highlight the opportunity for vitamin and mineral fortification policies for GFP and the necessity of nutrition guidelines for children with CD consuming a gluten-free diet.

Alimentary education should also become part of the therapeutic pathway to understand the importance of labels, choice of food, and combination of macro and micronutrients. Furthermore, the dietary therapeutic approach should encourage the use of naturally gluten-free products such as pseudo-cereals that have been shown to have good nutritional quality [20], and green vegetables, fruits, legumes, and fish [17,22,32]. 

A logical and intelligent strategy that could bring long-term benefits is the use of homemade products made with flours from other sources (other than rice and corn) complemented with proper nutrition education, including the avoidance of commercial GFP. Gluten restriction has important implications for nutrient adequacy, since staple foods (e.g., breads, pastas, and cereals) are key nutrient sources in the western diet, especially in young people. The use of nutrient-dense gluten-free flours, rather than starches, in homemade foods may improve nutrient intakes, and homemade recipes may allow for fat and sugar control. In this sense, chickpea flour registered the highest folate content followed by quinoa, amaranth, and flaxseed gluten-free flours; chickpea, flaxseed, and chia flours have higher protein contents; and tapioca, buckwheat, and brown rice flour are rich in carbohydrates and total starch [53]. The challenge in using alternative flours in natura in food preparation is the need for high food literacy (e.g., food skills, budgeting, and nutrition knowledge) and more time for meal planning and cooking in comparison to purchasing ready-to-serve products.

Regarding the limitations of the present study, it is important to state that there is a lack of nutritional information about gluten-free products on food composition since labels do not provide information on micronutrient contents. Data on salt and sodium intakes should be taken cautiously since we have not quantified discretionary salt use (salt added during cooking or at the table). Finally, the research is a follow up study of an initial project comparing dietary habits of children with and without CD, and the sample size was adapted to the comparison research. Therefore, sample representativeness error increases to 10% for conclusions drawn over the whole population of children and adolescents with CD and to 26% when concerning adolescents. Increasing the sample size in future studies will help to confirm the results found in here.

## 5. Conclusions

In summary, consumption of processed gluten-free products (GFP) is high in the Spanish celiac disease (CD) pediatric population, contributing to a large part of the energy, fat, saturated fat, and salt in their daily diet. At the same time, these GFP provide most of the fiber and carbohydrates needed to equilibrate a gluten-free diet. Children and adolescents with CD could benefit from reformulation of gluten-free commercial products, consisting of fat, saturated fat, salt reduction, and fiber enrichment. Moreover, families could also benefit from the empowerment to prepare nutritious homemade alternatives. Taken together, there is an opportunity to improve the health and nutritional status of the celiac population in Spain, a country with one of the highest prevalence of the disease in Europe, and with a gastronomic tradition of the Mediterranean diet, which could facilitate the adoption of a healthy diet while promoting cultural heritage.

## Figures and Tables

**Table 1:** Daily intake of processed and homemade cereal-based gluten-free products in Spanish children and adolescents with celiac disease.

	Total	Girls	Boys	Children ^1^	Adolescents ^2^
	(*n* = 70)	(*n* = 35)	(*n* = 35)	(*n* = 56)	(*n* = 14)
**Bread (processed) (g/day)**	59.2 ± 35.9	53.4 ± 36.1	65.1 ± 35.2	61.1 ± 36.2	52.0 ± 34.9
**Bread (homemade) (g/day)**	3.4 ± 11.1	4.0 ± 11.8	2.9 ± 10.5	2.2 ± 6.1	8.5 ± 21.5
**Breakfast cereals and cereal bars (processed)** **(g/day)**	11.8 ± 13.7	11.3 ± 12.7	12.4 ± 14.8	10.7 ± 11.5	16.2 ± 20.4
**Pasta (processed) (g/day)**	17.5 ± 18.1	15.0 ± 17.7	20.0 ± 18.5	16.7 ± 16.7	20.5 ± 23.6
**Fine bakery ware (processed) (g/day)**	38.1 ± 26.2	36.7 ± 21.9	39.6 ± 30.1	40.7 ± 26.6	27.9 ± 22.6
**Fine bakery ware (homemade) (g/day)**	6.8 ± 15.1	4.7 ± 12.9	8.8 ± 17.0	6.4 ± 13.9	7.9 ± 20.1
*Biscuits (processed) (g/day)*	18.7 ± 17.6	18.4 ± 18.1	18.9 ± 17.3	20.4 ± 17.5	11.7 ± 16.4
*Biscuits (homemade) (g/day)*	0.4 ± 2.5	0.2 ± 1.1	0.6 ± 3.4	0.4 ± 2.8	0.0 ± 0.0
*Pastry (processed) (g/day)*	19.8 ± 22.6	18.3 ± 21.8	21.4 ± 23.6	20.7 ± 23.8	16.2 ± 17.2
*Pastry (homemade) (g/day)*	6.4 ± 15.0	4.5 ± 12.7	8.2 ± 17.0	6.0 ± 13.7	7.9 ± 20.1
**Savoury cereal dishes (processed) (g/day)**	21.0 ± 30.1	13.2 ± 21.7	28.8 ± 35.3 *	19.4 ± 26.8	27.1 ± 41.5
**Savoury cereal dishes (homemade) (g/day) ^3^**	0.4 ± 3.2	0.8 ± 4.5	0.0 ± 0.0	0.5 ± 3.6	0.0 ± 0.0
*Pizza (processed) (g/day)*	10.3 ± 21.2	6.1 ± 16.1	14.5 ± 24.9	11.0 ± 21.2	7.6 ± 22.0
*Prepared food products (processed) (g/day)*	8.8 ± 17.7	5.7 ± 15.5	12.0 ± 19.4	6.2 ± 14.3	19.5 ± 25.3
*Snacks (processed) (g/day)*	1.4 ± 4.9	0.6 ± 2.4	2.3 ± 6.5	1.8 ± 5.5	0.0 ± 0.0 *
**Total processed GFP (g/day)**	147.9 ± 57.7	155.1 ± 60.0	140.7 ± 55.2	149.2 ± 57.6	142.6 ± 59.9
**Total homemade GFP (g/day)**	17.4 ± 32.9 ^#^	19.9 ± 33.8 ^#^	14.9 ± 32.3 ^#^	19.1 ± 35.2 ^#^	10.2 ± 21.1 ^#^

Data are expressed as average ± standard deviation. GFP: gluten-free products. ^1^ Children aged four to 12 years. ^2^ Adolescents aged 13 to 18 years. ^3^ Savory cereal dishes (homemade) include solely prepared food products (croquette). * *p* < 0.05 boys vs. girls or children vs. adolescents. ^#^ *p* < 0.05 processed vs. homemade.

**Table 2:** Contribution of gluten-free products, both processed and homemade, to the diet (energy and nutrient content) of Spanish children and adolescents, both sexes, with celiac disease.

	Total Daily Intake	Intake from GFP	% Contribution of GFP
	(*n* = 70)	(*n* = 70)	(*n* = 70)
Energy (kcal/day)	2051.0 ± 442.8	506.3 ± 170.5	25.1 ± 8.5
Fats (g/day)	93.4 ± 20.9	14.6 ± 7.3	16.2 ± 8.2
Saturated fat (g/day)	32.0 ± 7.9	5.4 ± 3.1	17.6 ± 9.5
Carbohydrates (g/day)	209.3 ± 66.0	83.3 ± 28.6	41.9 ± 16.0
Sugars (g/day)	89.2 ± 24.8	14.3 ± 7.2	16.7 ± 8.2
Protein (g/day)	77.5 ± 18.1	7.7 ± 3.3	10.3 ± 4.9
Fibre (g/day)	18.0 ± 7.6	5.7 ± 2.7	34.9 ± 18.0
Salt (g/day)	4.4 ± 2.3	1.4 ± 0.6	37.8 ± 22.4

Data are expressed as average ± standard deviation. GFP = gluten-free products.

**Table 3:** Contribution of gluten-free products to the diet (energy and nutrient content) of Spanish children and adolescents with celiac disease depending on gender and age.

	Total Daily Intake		Intake from GFP		% Contribution of GFP		Total Daily Intake		Intake from GFP		% Contribution of GFP	
	Boys	Girls	Boys	Girls	Boys	Girls	Children ^1^	Adolescents ^2^	Children ^1^	Adolescents ^2^	Children ^1^	Adolescents ^2^
	(*n* = 35)	(*n* = 35)	(*n* = 35)	(*n =* 35)	(*n =* 35)	(*n =* 35)	(*n =* 56)	(*n =* 14)	(*n =* 56)	(*n =* 14)	(*n =* 56)	(*n =* 14)
Energy (kcal)	2114 ± 294	1988 ± 550	562.7 ± 139.9	450.0 ± 181.3	27.1 ± 8.0	23.1 ± 8.6	2075 ± 451	1953 ± 408	513.6 ± 177.5	477.1 ± 140.7	25.2 ± 8.7	25.0 ± 8.0
Fats (g)	95.0 ± 16.17	91.9 ± 24.9	16.5 ± 7.1	12.8 ± 7.1	18.2 ± 9.2	14.2 ± 6.5	94.7 ± 21.2	94.7 ± 21.2	14.6 ± 7.4	14.6 ± 7.4	15.9 ± 7.9	15.9 ± 7.9
Saturated fat (g)	32.3 ± 5.8	31.7 ± 9.7	6.4 ± 3.1	4.5 ± 2.8	20.3 ± 10.2	14.8 * ± 8.1	32.3 ± 8.0	32.3 ± 8.0	5.5 ± 3.2	5.5 ± 3.2	17.8 ± 9.8	17.6 ± 9.8
Carbohydrates (g)	216.0 ± 40.2	202.6 ± 84.5	92.2 ± 23.9	74.3 ± 30.3	44.4 ± 15.0	39.5 ± 16.8	213.7 ± 70.0	191.8 ± 44.3	85.0 ± 28.8	76.3 ± 27.4	42.1 ± 16.1	41.2 ± 16.0
Sugars (g)	94.4 ± 24.1	84.0 ± 24.7	16.6 ± 7.6	12.0 ± 6.0	18.0 ± 7.9	15.3 ± 8.4	90.8 ± 24.5	82.7 ± 25.9	14.6 ± 7.3	13.0 ± 7.0	16.7 ± 8.1	16.5 ± 9.1
Protein (g)	80.2 ± 15.7	74.8 ± 20.0	8.6 ± 3.1	6.7 ± 3.4	11.1 ± 4.9	9.4 ± 4.9	78.1 ± 17.7	75.2 ± 19.9	7.8 ± 3.4	7.3 ± 3.1	10.2 ± 4.7	10.5 ± 6.1
Fibre (g)	18.4 ± 5.6	17.7 ± 9.2	6.1 ± 2.5	5.3 ± 2.8	36.7 ± 19.8	33.1 ± 16.2	18.2 ± 7.8	17.5 ± 7.0	5.8 ± 2.8	5.6 ± 2.3	34.7 ± 18.6	35.6 ± 16.1
Salt (g)	4.4 ± 1.4	4.4 ± 3.0	1.6 ± 0.5	1.3 ± 0.6	38.2 ± 16.0	37.3 ± 27.6	4.5 ± 2.5	4.2 ± 1.7	1.4 ± 0.6	1.5 ± 0.5	36.0 ± 18.6	44.8 ± 33.7

Data are expressed as average ± standard deviation. GFP = gluten free products. ^1^ Children aged four to 12 years. ^2^ Adolescents aged 13 to 18 years. * *p* < 0.05 boys vs. girls.

**Table 4:** Contribution of the different types of gluten-free products of to the daily energy intake in Spanish children and adolescents, both genders, with celiac disease.

	% Contribution to Total Energy Intake				
	Total	Girls	Boys	Children ^1^	Adolescents ^2^
	(*n* = 70)	(*n* = 35)	(*n* = 35)	(*n* = 56)	(*n* = 14)
Bread	8.4 ± 5.2 ^a^	8.0 ± 5.3 ^a^	8.9± 5.2 ^a^	8.6 ± 5.2 ^a^	7.7 ± 5.2 ^a^
Breakfast cereals and cereal bars	0.6 ± 0.7 ^b^	0.6 ± 0.7 ^b^	0.6 ± 0.7 ^b^	0.5 ± 0.6 ^b^	0.9 ± 1.0 ^b^
Pasta	3.1 ± 3.1 ^c^	2.7 ± 3.0 ^c^	3.4 ± 3.3 ^c^	3.1 ± 3.1 ^c^	3.1 ± 3.1 ^c^
Fine bakery ware	9.2 ± 6.0 ^a^	8.7 ± 5.0 ^a^	9.6 ± 6.8 ^a^	9.3 ± 5.8 ^a^	8.6 ± 6.8 ^a,d^
Savory cereal dishes	2.4 ± 3.3 ^c^	1.6 ± 2.7 ^b^	3.2 ± 3.7 *^,c^	2.1 ± 2.7 ^c^	3.4 ± 4.9 ^b,c,d^

Data are expressed as average ± standard deviation. ^1^ Children aged four to 12 years. ^2^ Adolescents aged 13 to 18 years. * *p* <0.05 boys vs. girls. Different letters denote statistically significant differences among types of gluten-free products.

**Table 5:** Contribution of the different types of gluten-free products of to the daily fat and saturated fat intakes in Spanish children and adolescents, both genders, with celiac disease.

	% Contribution to Total Fat Intake (% Contribution to Total Saturated Fat Intake)				
	Total	Girls	Boys	Children^1^	Adolescents ^2^
	(*n* = 70)	(*n* = 35)	(*n* = 35)	(*n* = 56)	(*n* = 14)
Bread	3.7 ± 2.7 ^a^	3.3 ± 2.2 ^a^	4.0 ± 3.2 ^a^	3.7 ± 2.9 ^a^	3.5 ± 2.3 ^a^
	(3.7 ± 3.4 ^e^)	(3.4 ± 2.6 ^e^)	(4.0 ± 4.1 ^e^)	(3.7 ± 3.5 ^e^)	(3.6 ± 3.1 ^e^)
Breakfast cereals and cereal bars	0.6 ± 1.2 ^b^	0.4 ± 0.8 ^b^	0.8 ± 1.5 ^b^	0.5 ± 1.0 ^b^	0.8 ± 1.8 ^b^
	(0.5 ±1.2 ^f^)	(0.3 ± 0.7 ^f^)	(0.8 ± 1.4 ^f^)	(0.5 ± 0.8 ^f^)	(0.8 ± 2.0 ^f^)
Pasta	0.4 ± 0.5 ^b^	0.3 ± 0.4 ^b^	0.5 ± 0.6 ^b^	0.4 ± 0.5 ^b^	0.6 ± 0.6 ^b^
	(0.5 ± 0.6 ^f^)	(0.4 ± 0.5 ^f^)	(0.5 ± 0.6 ^f^)	(0.4 ± 0.6 ^f^)	(0.6 ± 0.6 ^f^)
Fine bakery ware	9.6 ± 7.2 ^c^	8.8 ± 5.8 ^c^	10.5 ± 8.3 ^c^	9.6 ± 6.9 ^c^	10.1 ± 8.2 ^c^
	(10.7 ± 8.1 ^g^)	(9.6 ± 7.3 ^g^)	(11.8 ± 8.7 ^g^)	(11.1 ± 7.9 ^g^)	(9.3 ± 9.0 ^e^)
Savory cereal dishes	1.8 ± 2.8 ^d^	1.3 ± 2.2 ^b^	2.4 ± 3.2 ^d^	1.6 ± 2.4 ^d^	2.6 ± 4.2 ^a,b^
	(2.2 ± 4.0 ^h^)	(1.1 ± 2.1 ^f^)	(3.4 ± 5.0 *^,e^)	(2.0 ± 3.1 ^h^)	(3.3 ± 6.4 ^e,f^)

Data are expressed as average ± standard deviation. ^1^ Children aged four to 12 years. ^2^ Adolescents aged 13 to 18 years. * *p* < 0.05 boys vs. girls. Different letters denote statistically significant differences among types of gluten-free products (within total fat or saturated fat).

**Table 6:** Contribution of the different types of gluten-free products to the daily carbohydrate and sugar intakes in Spanish children and adolescents, both genders, with celiac disease.

	% Contribution to Total Carbohydrate Intake (% Contribution to Total Sugars Intake)				
	Total	Girls	Boys	Children ^1^	Adolescents ^2^
	(*n* = 70)	(*n* = 35)	(*n* = 35)	(*n* = 56)	(*n* = 14)
Bread	15.9 ± 10.7 ^a^	14.9 ± 10.1 ^a^	16.9 ± 11.2 ^a^	16.3 ± 10.5 ^a^	14.6 ± 11.6 ^a^
	(3.8 ± 3.5 ^e^)	(3.8 ± 3.6 ^e^)	(3.8 ± 3.5 ^e^)	(3.9 ± 3.8 ^e^)	(3.2 ± 2.1 ^e^)
Breakfast cereals and cereal bars	4.7 ± 5.6 ^b,c^	5.0 ± 6.1 ^b,c^	4.4 ± 5.1 ^b^	4.2 ± 4.8 ^b^	6.8 ± 7.9 ^b,c^
	(2.3 ± 3.8 ^f^)	(1.8 ± 3.8 ^f^)	(2.8 ± 3.8 ^e^)	(2.0 ± 2.8 ^f^)	(3.4 ± 6.4 ^e,f^)
Pasta	6.8 ± 7.2 ^b^	6.2 ± 7.1 ^b^	7.5 ± 7.4 ^b^	6.7 ± 7.1 ^c^	7.4 ± 7.9 ^b,c^
	(0.2 ± 0.2 ^g^)	(0.1 ± 0.2 ^g^)	(0.2 ± 0.2 ^f^)	(0.1 ± 0.2 ^g^)	(0.2 ± 0.3 ^f,g^)
Fine bakery ware	11.9 ± 7.8 ^a^	11.9 ± 7.3 ^a^	11.9 ± 8.4 ^a^	12.3 ± 7.4 ^a^	10.4± 9.4 ^a,c,d^
	(10.0 ± 7.4 ^h^)	(9.5 ± 6.7 ^h^)	(10.5 ± 8.0 ^f^)	(10.4 ± 7.4 ^h^)	(8.5 ± 7.2 ^e,g^)
Savory cereal dishes	3.3 ± 4.8 ^c^	2.1 ± 4.1 ^c^	4.4 ± 5.1 *^,c^	2.8 ± 3.7 ^b^	5.2 ± 7.6 ^b,d^
	(0.7 ± 1.5 ^i^)	(0.4 ± 0.9 ^g^)	(1.0 ± 1.8 *^,g^)	(0.6 ± 1.0 ^i^)	(1.3 ± 2.5 ^f^)

Data are expressed as average ± standard deviation. ^1^ Children aged four to 12 years. ^2^ Adolescents aged 13 to 18 years. * *p* < 0.05 boys vs. girls. Different letters denote statistically significant differences among types of gluten-free products (within total carbohydrate or sugars).

**Table 7:** Contribution of the different types of gluten-free products of to the daily protein intake in Spanish children and adolescents, both genders, with celiac disease.

	% Contribution to Total Protein Intake				
	Total	Girls	Boys	Children ^1^	Adolescents ^2^
	(*n* = 70)	(*n* = 35)	(*n* = 35)	(*n* = 56)	(*n* = 14)
Bread	3.0 ± 2.6 ^a^	3.3 ± 3.3 ^a^	2.7 ± 1.7 ^a^	3.0 ± 2.7 ^a^	3.0 ± 2.4 ^a^
Breakfast cereals and cereal bars	1.1 ± 1.3 ^b^	1.2 ± 1.4 ^b^	1.1 ± 1.2 ^b^	1.0 ± 1.1 ^b^	1.7 ± 2.0 ^a^
Pasta	1.5 ± 1.6 ^b^	1.3 ± 1.6 ^b^	1.7 ±1.6 ^b^	1.5 ± 1.6 ^b^	1.5 ± 1.5 ^a^
Fine bakery ware	2.7 ± 2.0 ^a^	2.3 ± 1.5 ^a, c^	3.1 ± 2.3 ^a^	2.8 ± 2.0 ^a^	2.2 ± 2.0 ^a^
Savory cereal dishes	2.0 ± 3.4 ^b^	1.3 ± 2.2 ^b, c^	2.6 ± 4.2 ^a, b^	1.9 ± 2.6 ^b^	2.5 ± 5.6 ^a^

Data are expressed as average ± standard deviation. ^1^ Children aged four to 12 years. ^2^ Adolescents aged 13 to 18 years. Different letters denote statistically significant differences among types of gluten-free products.

**Table 8:** Contribution of the different types of gluten-free products of to the daily fiber intake in Spanish children and adolescents, both genders, with celiac disease.

	% Contribution to Total Fiber Intake				
	Total	Girls	Boys	Children ^1^	Adolescents ^2^
	(*n* = 70)	(*n* = 35)	(*n* = 35)	(*n* = 56)	(*n* = 14)
Bread	20.1 ± 15.0 ^a^	19.3 ± 13.0 ^a^	20.9 ± 16.9 ^a^	20.6 ± 15.7 ^a^	18.1 ± 11.7 ^a^
Breakfast cereals and cereal bars	3.9 ± 6.2 ^b^	3.9 ± 4.6 ^b^	3.9 ± 7.6 ^b^	3.1 ± 3.8 ^b^	7.1 ± 11.5 ^b^
Pasta	3.1 ± 3.8 ^b^	2.8 ± 3.5 ^b, d^	3.4 ± 4.1 ^b^	3.0 ± 3.9 ^b^	3.4 ± 3.4 ^b^
Fine bakery ware	6.5 ± 5.3 ^c^	6.6 ± 5.1 ^c^	6.3 ± 5.6 ^c^	7.0 ± 4.9 ^c^	4.7 ± 6.7 *^,b^
Savory cereal dishes	2.4 ± 4.6 ^b^	1.5 ± 3.8 ^d^	3.4 ± 5.2 ^b^	1.9 ± 3.7 ^b^	4.5 ± 6.9 ^b^

Data are expressed as average ± standard deviation. ^1^ Children aged four to 12 years. ^2^ Adolescents aged 13 to 18 years. * *p* < 0.05 adolescents vs. children. Different letters denote statistically significant differences among types of gluten-free products.

**Table 9:** Contribution of the different types of gluten-free products of to the daily salt intake in Spanish children and adolescents, both genders, with celiac disease.

	% Contribution to Total Salt Intake				
	Total	Girls	Boys	Children ^1^	Adolescents ^2^
	(*n* = 70)	(*n* = 35)	(*n* = 35)	(*n* = 56)	(*n* = 14)
Bread	20.5 ± 17.8 ^a^	21.1 ± 21.3 ^a^	19.9 ± 13.7 ^a^	19.4 ± 13.8 ^a^	24.6 ± 29.1 ^a^
Breakfast cereals and cereal bars	3.2 ± 4.8 ^b^	4.0 ± 5.5 ^b^	2.5 ± 3.8 ^b^	2.6 ± 3.8 ^b^	5.7 ± 7.3 ^b^
Pasta	0.5 ± 1.4 ^c^	0.3 ± 0.5 ^c^	0.8 ± 1.8 ^c^	0.4 ± 1.1 ^c^	1.1 ± 2.1 ^b^
Fine bakery ware	9.3 ± 8.5 ^d^	9.8 ± 9.6 ^d^	8.8 ± 7.4 ^d^	10.0 ± 8.8 ^d^	6.5 ± 7.1 ^b^
Savory cereal dishes	6.2 ± 11.7 ^b^	5.1 ± 13.9 ^b^	7.3 ± 9.2 *^,d^	4.7 ± 7.3 ^b^	12.0 ± 21.5 ^b^

Data are expressed as average ± standard deviation. ^1^ Children aged four to 12 years. ^2^ Adolescents aged 13 to 18 years. * *p* < 0.05 boys vs. girls. Different letters denote statistically significant differences among types of gluten-free products.

## Data Availability

The data on gluten-free food composition used in this study are openly available in the institutional repository at Universidad San PabloCEU [CEU Repositorio Institucional] at http://hdl.handle.net/10637/13562. The data on dietary intakes presented in this study are available on request from the corresponding author. Last accessed on 14 April 2022.

## References

[1] Polanco I. (2008). Libro Blanco de la Enfermedad Celiaca.

[2] King J.A., Jeong J., Underwood F.E., Quan J., Panaccione N., Windsor J.W., Coward S., de Bruyn J., Ronksley P.E., Shaheen A.A. (2020). Incidence of Celiac Disease Is Increasing Over Time: A Systematic Review and Meta-analysis. Am. J. Gastroenterol..

[3] Mahadev S., Murray J.A., Wu T.-T., Chandan V.S., Torbenson M.S., Kelly C.P., Maki M., Green P.H.R., Adelman D., Lebwohl B. (2017). Factors associated with villus atrophy in symptomatic coeliac disease patients on a gluten-free diet. Aliment. Pharm. Ther..

[4] Lebwohl B., Murray J.A., Rubio-Tapia A., Green P.H.R., Ludvigsson J.F. (2014). Predictors of persistent villous atrophy in coeliac disease: A population-based study. Aliment. Pharmacol. Ther..

[5] Verrill L., Zhang Y., Kane R.J. (2013). Food label usage and reported difficulty with following a gluten-free diet among individuals in the USA with coeliac disease and those with non-coeliac gluten sensitivity. Hum. Nutr. Diet..

[6] Thompson T., Dennis M., Higgins L.A., Lee A.R., Sharrett M.K. (2005). Gluten-free diet survey: Are Americans with coeliac disease consuming recommended amounts of fibre, iron, calcium and grain foods?. J. Hum. Nutr. Diet..

[7] Ferrara P., Cicala M., Tiberi E., Spadaccio C., Marcella L., Gatto A., Calzolari P., Castellucci G. (2009). High fat consumption in children with celiac disease. Acta Gastro Enterol. Belg..

[8] Mariani P., Viti M.G., Montouri M., La Vecchia A., Cipolletta E., Calvani L., Bonamico M. (1998). The Gluten-Free Diet: A Nutritional Risk Factor for Adolescents with Celiac Disease?. J. Pediatr. Gastroenterol. Nutr..

[9] Hopman E.G., Le Cessie S., Von Blomberg B.M.E., Mearin M.L. (2006). Nutritional Management of the Gluten free Diet in Young People with Celiac Disease in The Netherlands. J. Pediatr. Gastroenterol. Nutr..

[10] Öhlund K., Olsson C., Hernell O., Öhlund I. (2010). Dietary shortcomings in children on a gluten-free diet. J. Hum. Nutr. Diet..

[11] Kautto E., Ivarsson A., Norstrom F., Hogberg L., Carlsson A., Hornell A. (2014). Nutrient intake in adolescent girls and boys diagnosed with coeliac disease at an early age is mostly comparable to their non-coeliac contemporaries. J. Hum. Nutr. Diet..

[12] Salazar Quero J.C., Espín Jaime B., Rodríguez Martínez A., Argüelles Martín F., García Jiménez R., Rubio Murillo M., Martín A.P. (2015). Nutritional assessment of gluten-free diet. Is gluten-free diet deficient in some nutrient?. An. Pediatr..

[13] Zuccotti G., Fabiano V., Dilillo D., Picca M., Cravidi C., Brambilla P. (2013). Intakes of nutrients in Italian children with celiac disease and the role of commercially available gluten-free products. J. Hum. Nutr. Diet..

[14] Babio N., Alcázar M., Castillejo G., Recasens M., Martínez-Cerezo F., Gutiérrez-Pensado V., Masip G., Vaqué C., Vila-Martí A., Torres-Moreno M. (2017). Patients with Celiac Disease Reported Higher Consumption of Added Sugar and Total Fat Than Healthy Individuals. J. Pediatr. Gastroenterol. Nutr..

[15] Sue A., Dehlsen K., Ooi C.Y. (2018). Paediatric Patients with Coeliac Disease on a Gluten-Free Diet: Nutritional Adequacy and Macro- and Micronutrient Imbalances. Curr. Gastroenterol. Rep..

[16] Larretxi I., Simon E., Benjumea L., Miranda J., Bustamante M.A., Lasa A., Eizaguirre F.J., Churruca I. (2019). Gluten-free-rendered products contribute to imbalanced diets in children and adolescents with celiac disease. Eur. J. Nutr..

[17] Di Nardo G., Villa M.P., Conti L., Ranucci G., Pacchiarotti C., Principessa L., Parisi P. (2019). Nutritional Deficiencies in Children with Celiac Disease Resulting from a Gluten-Free Diet: A Systematic Review. Nutrients.

[18] Ballestero-Fernández CVarela-Moreiras G., Úbeda N., Alonso-Aperte E. (2019). Nutritional Status in Spanish Children and Adolescents with Celiac Disease on a Gluten Free Diet Compared to Non-Celiac Disease Controls. Nutrients.

[19] Nestares T., Martín-Masot R., Labella A., Aparicio V.A., Flor-Alemany M., López-Frías M., Maldonado J. (2020). Is a Gluten-Free Diet Enough to Maintain Correct Micronutrients Status in Young Patients with Celiac Disease?. Nutrients.

[20] Vici G., Belli L., Biondi M., Polzonetti V. (2016). Gluten free diet and nutrient deficiencies: A review. Clin. Nutr..

[21] Kreutz J.M., Adriaanse M.P., van der Ploeg E.M., Vreugdenhil A.C. (2020). Narrative Review: Nutrient Deficiencies in Adults and Children with Treated and Untreated Celiac Disease. Nutrients.

[22] Kozioł-Kozakowska A., Salamon D., Grzenda-Adamek Z., Krawczyk A., Duplaga M., Gosiewski T., Kowalska-Duplaga K. (2021). Changes in Diet and Anthropometric Parameters in Children and Adolescents with Celiac Disease-One Year of Follow-Up. Nutrients.

[23] Lionetti E., Antonucci N., Marinelli M., Bartolomei B., Franceschini E., Gatti S., Catassi G.N., Verma A.K., Monachesi C., Catassi C. (2020). Nutritional Status, Dietary Intake, and Adherence to the Mediterranean Diet of Children with Celiac Disease on a Gluten-Free Diet: A Case-Control Prospective Study. Nutrients.

[24] Ting A., Katz T., Sutherland R., Liu V., Tong C.W., Gao Y., Lemberg D.A., Krishnan U., Gupta N., Coffey M.J. (2020). Evaluating the Dietary Intakes of Energy, Macronutrients, Sugar, Fibre, and Micronutrients in Children with Celiac Disease. J. Pediatr. Gastroenterol. Nutr..

[25] Melini V., Melini F. (2019). Gluten-Free Diet: Gaps and Needs for a Healthier Diet. Nutrients.

[26] Taetzsch A., Das S.K., Brown C., Krauss A., Silver R.E., Roberts S.B. (2018). Are gluten-free diets more nutritious? An evaluation of self-selected and recommended gluten-free and gluten-containing dietary patterns. Nutrients.

[27] Miranda J., Lasa A., Bustamante M.A., Churruca I., Simon E. (2014). Nutritional differences between a gluten-free diet and a diet containing equivalent products with gluten. Plant Foods Hum. Nutr..

[28] Fry L., Madden A., Fallaize R. (2018). An investigation into the nutritional composition and cost of gluten-free versus regular food products in the UK. J. Hum. Nutr. Diet..

[29] Elliott C. (2018). The Nutritional Quality of Gluten-Free Products for Children. Pediatrics.

[30] Calvo-Lerma J., Crespo-Escobar P., Martínez-Barona S., Fornés-Ferrer V., Donat E., Ribes-Koninckx C. (2019). Differences in the macronutrient and dietary fibre profile of gluten-free products as compared to their gluten-containing counterparts. Eur. J. Clin. Nutr..

[31] Fajardo V., González M.P., Martínez M., Samaniego-Vaesken M.L., Achón M., Úbeda N., Alonso-Aperte E. (2020). Updated Food Composition Database for Cereal-Based Gluten Free Products in Spain: Is Reformulation Moving on?. Nutrients.

[32] Pellegrini N., Agostoni C. (2015). Nutritional aspects of gluten-free products. J. Sci. Food Agric..

[33] Cyrkot S., Anders S., Kamprath C., Liu A., Mileski H., Dowhaniuk J., Nasser R., Marcon M., Brill H., Turner J.M. (2020). Folate content of gluten-free food purchases and dietary intake are low in children with coeliac disease. Int. J. Food Sci. Nutr..

[34] Jamieson J.A., Weir M., Gougeon L. (2018). Canadian packaged gluten-free foods are less nutritious than their regular gluten-containing counterparts. PeerJ.

[35] Larretxi I., Txurruka I., Navarro V., Lasa A., Bustamante M.Á., Fernández-Gil M.d.P., Simón E., Miranda J. (2019). Micronutrient Analysis of Gluten-Free Products: Their Low Content Is Not Involved in Gluten-Free Diet Imbalance in a Cohort of Celiac Children and Adolescent. Foods.

[36] Allen B., Orfila C. (2018). The Availability and Nutritional Adequacy of Gluten-Free Bread and Pasta. Nutrients.

[37] Jastrebova J., Jägerstad M., Preedy V.R., Srirajaskanthan R., Patel V. (2013). Novel fortification strategies for staple gluten-free products. Handbook of Food Fortification and Health: From Concepts to Public Health Applications.

[38] Barone M., Della Valle N., Rosania R., Facciorusso A., Trotta A., Cantatore F.P., Falco S., Pignatiello S., Viggiani M.T., Amoruso A. (2016). A comparison of the nutritional status between adult celiac patients on a long-term, strictly gluten-free diet and healthy subjects. Eur. J. Clin. Nutr..

[39] Larretxi I., Churruca I., Navarro V., Miranda J., Lasa A., Bustamante M.A., Simon E. (2020). Effect of analytically measured fibre and resistant starch from gluten-free products on the diets of individuals with celiac disease. Nutrition.

[40] European Food Safety Authority (2014). Guidance on the EU Menu methodology. EFSA J..

[41] Fajardo V., González M.P., Martínez M., Samaniego Vaesken M.d.L., Achón M., Úbeda N., Alonso-Aperte E. Gluten Free Food Composition Database (GLUTENFREE-2019). CEU Rel (Institutional Repository). http://hdl.handle.net/10637/13562.

[42] Ruiz E., Ávila J.M., Valero T., Del Pozo S., Rodriguez P., Aranceta-Bartrina J., Gil Á., González-Gross M., Ortega R.M., Serra-Majem L. (2016). Macronutrient Distribution and Dietary Sources in the Spanish Population: Findings from the ANIBES Study. Nutrients.

[43] Bartrina J.A., Val V.A., Aldalur E.M., Muñoz E.M.d., Anta R.M.O., Pérez-Rodrigo C., Izquierdo J.Q., Martín A.R., Viñas B.R., Grupo Colaborativo de la Sociedad Española de Nutrición Comunitaria (SENC) (2016). Guías alimentarias para la población española (SENC, diciembre 2016); la nueva pirámide de la alimentación saludable. Nutr. Hosp..

[44] Penagini F., DiLillo D., Meneghin F., Mameli C., Fabiano V., Zuccotti G.V. (2013). Gluten-Free Diet in Children: An Approach to a Nutritionally Adequate and Balanced Diet. Nutrients.

[45] European Food Safety Authority Panel on Dietetic Products, Nutrition, and Allergies (NDA) (2010). Scientific Opinion on Dietary Reference Values for carbohydrates and dietary fibre. EFSA J..

[46] Valletta E., Fornaro M., Cipolli M., Conte S., Bissolo F., Danchielli C. (2010). Celiac disease and obesity: Need for nutritional follow-up after diagnosis. Eur. J. Clin. Nutr..

[47] Capriati T., Francavilla R., Ferretti F., Castellaneta S., Ancinelli M., Diamanti A. (2016). The overweight: A rare presentation of celiac disease. Eur. J. Clin. Nutr..

[48] Anania C., Pacifico L., Olivero F., Perla F.M., Chiesa C. (2017). Cardiometabolic risk factors in children with celiac disease on a gluten-free diet. World J. Clin. Pediatr..

[49] Turck D., Bohn T., Castenmiller J., de Henauw S., Hirsch-Ernst K.I., Knutsen H.K., Maciuk A., Mangelsdorf I., McArdle H.J., European Food Safety Authority Panel on Dietetic Products, Nutrition, and Allergies (NDA) (2022). Scientific Opinion on the tolerable upper intake level for dietary sugars. EFSA J..

[50] Partearroyo T., Samaniego-Vaesken M.d.L., Ruiz E., Aranceta-Bartrina J., Gil Á., González-Gross M., Ortega R.M., Serra-Majem L., Varela-Moreiras G. (2019). Sodium Intake from Foods Exceeds Recommended Limits in the Spanish Population: The ANIBES Study. Nutrients.

[51] AECOSAN, Ministerio de Sanidad, Consumo y Bienestar Social (2020). Collaboration Plan for the Improvement of the Composition of Food and Beverages and Other Measures. https://www.aesan.gob.es/AECOSAN/docs/documentos/nutricion/Plan_Colaboracion_INGLES.pdf.

[52] Royal Decree Law No 308/2019, of 26 April 2019, by which the quality standard for bread is approved. Ministry of the Presidency, Relations with the Courts and Equality. Official Newsletter of the State No113, of 11 May 2019. https://www.boe.es/eli/es/rd/2019/04/26/308.

[53] Jamieson J.A., Viana L., English M.M. (2020). Folate Content and Chemical Composition of Commercially Available Gluten-Free Flour Alternatives. Plant Foods Hum. Nutr..

